# Cyclin D1 over expression as a prognostic factor in patients with tobacco-related intraoral squamous cell carcinoma

**Published:** 2011-04

**Authors:** Ashok M. Shenoy

**Affiliations:** Department of Head & Neck Surgery, Kidwai Memorial Institute of Oncology, Banglore 560 029, India profshenoy@gmail.com

Das *et al*^1^ in this issue investigated the molecular profile of the tobacco related oral squamous cell carcinoma (OSCC) and established a clear link between the marker cyclin D1 expression. Their findings have implications in the understanding of the biology of the ubiquitous OSCC seen abundantly in the Indian Subcontinent. It is well established that certain molecular imprints at the cellular level precede the development of the invasive squamous cancer and molecular mutations are involved in the progression from normal to dysplasia to carcinoma *in situ* to invasive cancer; it is not the specific order of events but the final accumulation of events that culminate in the phenotypic manifestation of a malignant tumour in the vulnerable host^2^. Hence, while the development of OSCC is perceived commonly by clinicians as a continuum, there is no predictable step-wise progression that helps in the detection and arrest of the carcinogenic event at any point in the precancerous stage/s. Further, these molecular events can occur in seemingly normal appearing mucosa which further confounds the problems of secondary prevention or controlling the process at the pre-malignant stage.

Abnormal expression of cyclin D1 and cyclin-dependent kinases (CDKs) have been considered to be one of the most important factors in the tumourigenesis of various human malignancies. Amplification of the cyclin D1 gene was shown to be occurring in early stage of head and neck cancer and significantly associated with high proliferative activity^1^. This study is a sequel to the earlier research contributions of this team where the authors have shown that aneuploidy and higher S phase fraction in metastatic tumour cells of oral carcinoma were associated with poor prognosis and low disease-free survival. In this study, they have gone one step ahead and linked the overexpressed cyclin D1 with the cell cycle correlates of proliferative activity specifically aneuploidy and the S phase growth factions. They have also associated this molecular marker with significantly more aggressive course in terms of adverse clinicopathological features in the patients and tumours as well. This study documented significantly higher frequency of overexpression of cyclin D1 in patients with advanced age, advanced tumours stage and lymph node metastasis. Further, relatively higher frequency of cyclin D1 immunoreactivity was also seen in patients with less differentiated tumours suggesting inverse correlation of cyclin D1 expression with histological differentiation of tumour.

The impact of incorporating cyclin D1 analysis in the future decision making paradigm in the management of OSCC has several benefits. First and foremost the ‘identification and labelling’ of malignant OSCC as less biologic grade and amenable to curative treatment can permit the health care providers to provide the most optimal treatment at ‘all costs’. To be more specific in this era of treatment individualization, it may not be too distant future to enable and aid the clinicians to stratify and integrate the overall risk factors with respect to disease molecular profile to the traditional host and tumour staging (the significant ones *viz*., TP53, cyclin D1 antisense, EGFR overexpression, HPV genome in tumour tissue) and optimize treatment strategies in the treatment naive individuals. Finally, it is hoped to help in identifying those individuals who would benefit from chemoradiotherapy with organ preservation (OP) in contrast to those who are ‘better off’ with primary surgical management. Reports coming about HPV related oropharyngeal and laryngeal supraglottic cancers^3^,^4^ indicate that minor mutagenic ‘events’ caused by HPV in individuals with this type of carcinogenesis have a very favourable outcome to only external beam radiotherapy(EBRT). On the contrary, Temam *et al*^5^ noted that those tumors that were associated with PT53 overexpression did not lend themselves to a favourable outcome after neoadjuvant chemotherapy. Alsner *et al*^6^ found similar results with respect to EBRT thus preventing successful OP and disease control. In other words, it may be possible to incorporate biologic parameters at the pre-treatment level into the TNM and patient parameters to make the optimal treatment delivery with minimization of both the cost and morbidity of the treatment.

But to make it practical, this test has to be standardized at all medical centers and more importantly be inexpensive and expeditious. This should be the natural progression and culmination of these research methodologies whereby more and more centers in the country can with the wealth of clinical material undertake multicentric trials under the aegis of a governmental national agency to characterize conclusively the molecular correlates of tumour behaviour (both favourable and adverse) to commonly used treatment modalities and help individualize treatment protocols with better oncologic and functional outcomes.
